# Supplementary material for: Heteronemin and Tetrac Induce Anti-Proliferation by Blocking EGFR-Mediated Signaling in Colorectal Cancer Cells
Source: Mar Drugs. 2022 Jul 27;20(8):482. doi: 10.3390/md20080482 (PMC9410344; doi:10.3390/md20080482)
Supplement: Supplementary file 1 [file marinedrugs-20-00482-s001.zip › marinedrugs-1812412-supplementary.pdf]

Supplementary information for

Heteronemin and Tetrac Induce Anti-proliferation by Blocking EGFR-mediated Signaling in Colorectal Cancer Cells

Sukanya Unson[1], Tung-Cheng Chang[2], Yung-Ning Yang[3], Shwu-Huey Wang[4], Chi-Hung Huang[5], Dana R. Crawford[6], Haw-Ming Huang[7], Zi-Lin Li[8,9], Hung-Yun Lin [9,10,11,12,13*], Jacqueline Whang-Peng[12*], Kuan Wang[3], Paul J. Davis[13, 14], Wen-Shan Li[15, 16]

Correspondence to: jqwpeng@nhri.edu.tw and linhy@tmu.edu.tw

This PDF file includes

Supplementary figures S1 and S2.

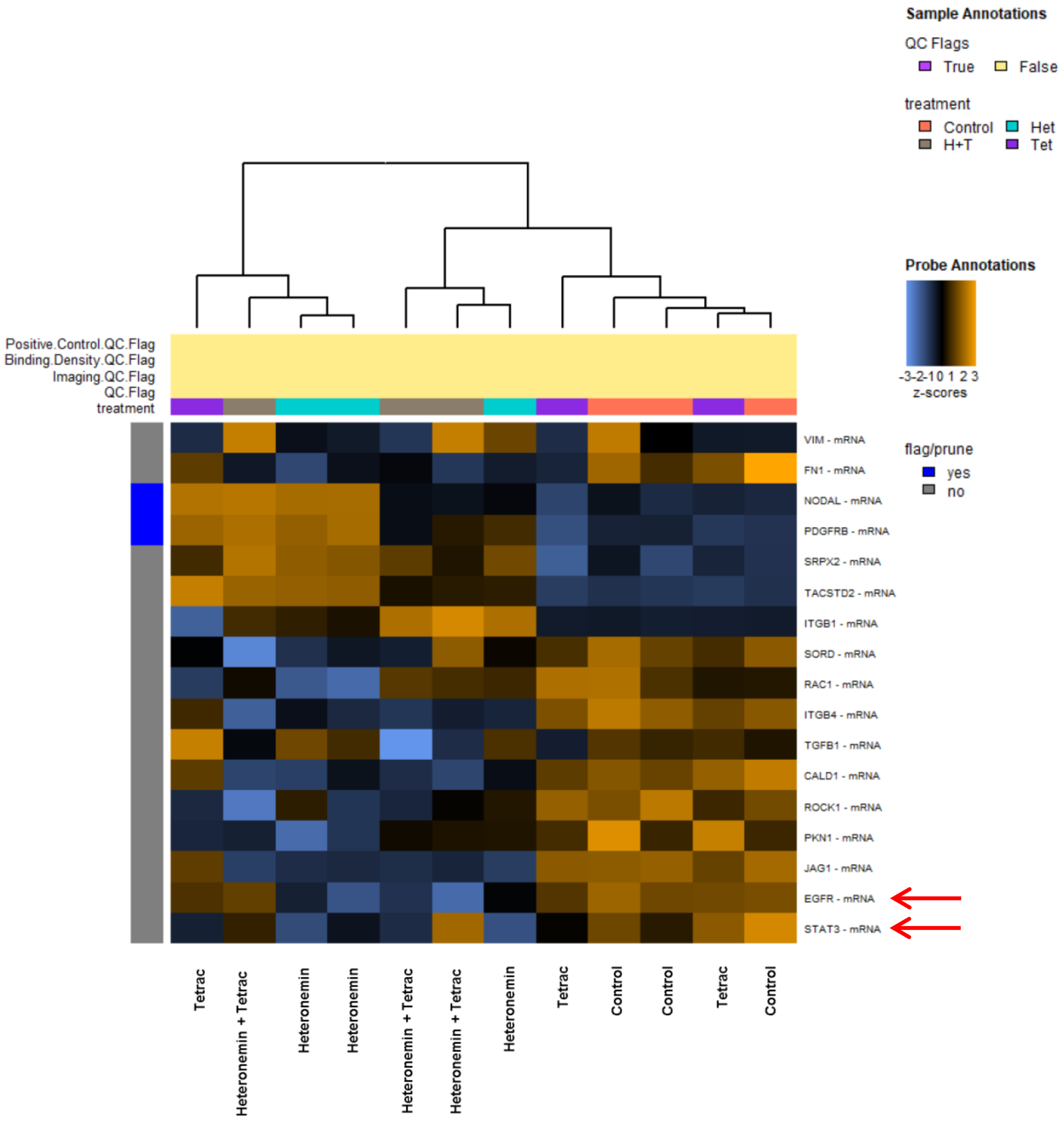

Figure S1. Heatmap of cell motility genes that showed significant ($p < 0.05$) upregulation or downregulation. HCT-116 cells were treated with heteronemin, tetrac, and their combination for 24 h. mRNA expression levels were identified by a NanoString® analysis using the nCounter PanCancer Progression Panel.

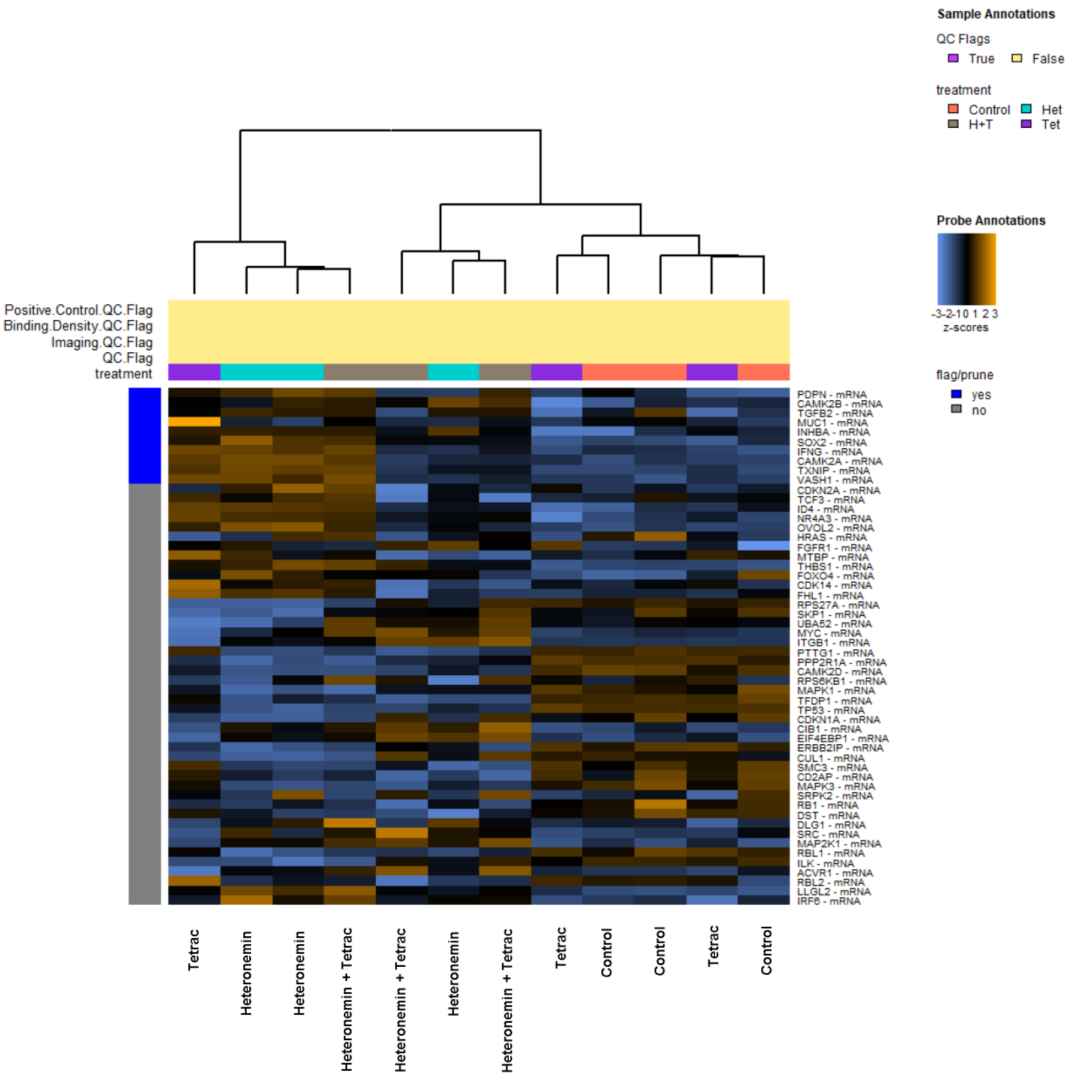

Figure S2. Heatmap of gene expressions involved in the cell cycle showed significant (p < 0.05) upregulation or downregulation. HCT-116 cells were treated with heteronemin, tetrac, and their combination for 24 h. mRNA expressions were identified by NanoString® analysis using the nCounter PanCancer Progression Panel.
